# Prognostic impact of additive chemotherapy after curative resection of metachronous colorectal liver metastasis: a single-centre retrospective study

**DOI:** 10.1186/s12885-021-07941-2

**Published:** 2021-05-03

**Authors:** Matthias Kelm, Julia Schollbach, Friedrich Anger, Armin Wiegering, Ingo Klein, Christoph-Thomas Germer, Nicolas Schlegel, Volker Kunzmann, Stefan Löb

**Affiliations:** 1 Department of General, Visceral, Transplant, Vascular and Pediatric Surgery, University Hospital of Würzburg, Oberdürrbacherstr. 6, 97080 Würzburg, Germany; 2 Theodor-Boveri-Institute, Biocenter, University of Würzburg, Am Hubland, 97074 Würzburg, Germany; 3 Comprehensive Cancer Center Mainfranken, University of Würzburg, Josef-Schneider-Str. 6, 97080 Würzburg, Germany; 4 Department of Internal Medicine II, University of Würzburg, Würzburg, Germany

**Keywords:** Colorectal liver metastasis, Colorectal cancer, Additive chemotherapy, Hepatobiliary surgery, Metachronous liver metastasis, Surgical oncology

## Abstract

**Background:**

A prognostic benefit of additive chemotherapy in patients following resection of metachronous colorectal liver metastases (CRLM) remains controversial. Therefore, the goal of this retrospective study was to investigate the impact of perioperative chemotherapy on disease-free survival (DFS) and overall survival (OS) of patients after curative resection of metachronous CRLM.

**Methods:**

In a retrospective single-centre study, patients after curative resection of metachronous CRLM were included and analysed for DFS and OS with regard to the administration of additive chemotherapy. The Kaplan-Meier method was applied to compare DFS and OS while Cox regression models were used to identify independent prognostic variables.

**Results:**

Thirty-four of 75 patients were treated with additive 5-FU based chemotherapy. OS was significantly prolonged in this patient subgroup (62 vs 57 months; *p* = 0.032). Additive chemotherapy significantly improved 10-year survival rates (42% vs 0%, *p* = 0.023), but not 5-year survival (58% vs 42%, *p* = 0.24). Multivariate analysis identified additive chemotherapy (*p* = 0.016, HR 0.44, 95% CI 0.23–0.86), more than five CRLM (*p* = 0.026, HR 2.46, 95% CI 1.16–10.32) and disease recurrence (0.009, HR 2.70, 95% CI 1.29–5.65) as independent risk factors for OS.

**Conclusion:**

Additive chemotherapy significantly prolonged OS and 10-year survival in patients after curative resection of metachronous CRLM. Randomized clinical trials are needed in the future to identify optimal chemotherapy regimens for those patients.

## Background

Colorectal cancer (CRC) is one of the most common malignancies worldwide with an increasing incidence especially in young adults [1]. Up to 50% of patients with CRC develop distant metastases during their lifetime of which the liver represents the primary site [2–6]. However, only 20% of patients with CRLM are defined as potentially resectable [7–9]. Multimodal treatment protocols including chemotherapy and surgical resection of CRLM are the only chance of cure for these patients and have resulted in five-year overall survival rates of up to 50% in the past [10–12]. Continuous advancements in chemotherapeutic regimens and the introduction of intensified multimodal protocols have effectively improved prognosis of CRLM patients.

Treatment guidelines for synchronous CRLM are well established [13] and summarized in a consensus statement by the Expert Group on OncoSugery management of Liver Metastases (EGOSLIM group) [14]. However, while treatment recommendations for patients with metachronous disease remain heterogeneous according to different national guidelines, it gets even more inconsistent in terms of additive chemotherapy. While the National Comprehensive Cancer Networks (NCCN) specifically recommends additive chemotherapy for metachronous CRLM [15, 16], European Society of Medical Oncology (ESMO) guidelines do not recommend routine additive chemotherapy after curative resection of primarily resectable metachronous CRLM [17]. Only in case of unfavourable prognostic criteria (e.g. FONG-score, [18]) or in case the patient did not receive any previous systemic therapy for metastatic disease, additive chemotherapy can be administered upon an individual decision [17].

While some studies have demonstrated a significantly improved disease-free survival (DFS) after additive chemotherapy (CTx) for patients with CRLM, statistical significance was missing for patients’ OS despite a clearly prolonged median OS [19, 20]. In addition, most of the current evidence regarding CRLM includes patients with synchronous and metachronous CRLM resulting in heterogeneous patient cohorts [21–23]. Thus, mixing patients with synchronous and metachronous CRLM limits the conclusion drawn from the available data with regard to additive perioperative treatment, since clinical outcomes as well as molecular investigations suggest biological differences between both subtypes of CRLM [24, 25]. Therefore, the objective of this single-centre study was to investigate and evaluate the prognostic impact of additive chemotherapy in patients after curative resection of metachronous CRLM.

## Methods

### Study population

This is a retrospective single centre study. Among all patients who underwent a first hepatectomy for CRLM between January 2003 and December 2016 at the Department of Surgery at the University Hospital of Wuerzburg, 101 patients with metachronous colorectal liver metastasis who underwent macroscopic radical liver resection were identified. Patients with synchronous CRLM, extrahepatic disease or R1/2 resections were excluded from the study. No patient underwent concomitant local-ablative therapy or staged hepatectomies.

Metachronous CRLM were defined as metastasis detected after curative treatment of the primary tumour and divided into early-onset (< 12 months) (EM-CRLM) and late-onset (> 12 months) metachronous CRLM (LM-CRLM). The extent of liver disease was routinely assessed by CT scan (3-phase), and, if considered necessary, additionally via MRI scans. Extrahepatic disease was ruled out by CT scans of abdomen and thorax. Follow-up assessment of the primary tumour was done in accordance with the national S3-guideline [26].

Sociodemographic (age, sex) as well as clinicopathological data including the site of the primary tumour, its lymph node status as well as number and size of CLRM and preoperative carcinoembryonic antigen (CEA) serum levels were collected for each patient. Major liver resection was defined as removal of three or more liver segments. R0 resection was defined as complete removal of the tumour with negative surgical margins (> 1 mm). Surgical margins of less than 1 mm were defined as R1 resection whereas macroscopically incomplete resection was referred to as R2 resection. Recurrent disease was classified as hepatic or extrahepatic by the initial site of onset.

### Chemotherapy

Chemotherapy regimens were assessed for every patient including additive and perioperative CTx. Indication for additive CTx was made in specialized interdisciplinary tumor conferences on an individual basis and, if recommended, additive CTx was started within 3 months after liver resection according to the current national guidelines. Standard therapies were fluorouracil-based with mainly FOLFOX (FOLFOX4 or FOLFOX6) for a duration of 3 to 6 months. Other regimens such as FOLFIRI, CAPOX or Capecitabine were given according to the general condition of the patient, tolerance to Oxaliplatin or preferences of the medical oncologist. Successful administration of additive chemotherapy was defined as at least 3 months’ treatment. In case of primarily non-resectable hepatic disease, 2 to 4 months of 5-FU based (FOLFOX, FOLFIRI) neoadjuvant chemotherapy including monoclonal antibodies according to the individual KRAS-status (Cetuximab, Bevacizumab, Panitumumab) were given. The remaining doses to complete 6 months of perioperative chemotherapy in total were administered postoperatively as additive chemotherapy.

### Statistical analysis

Descriptive data were presented as median with range or total with percent. Differences in patient characteristics were assessed by Chi-square, Fisher’s exact or Mann-Whitney-U in accordance to the data scale and distribution. Overall survival (OS) as well as Disease Free Survival (DFS) were determined using the Kaplan-Meier method and compared with the log-rank test. OS and DFS were defined as the time from hepatic resection to the time of death or initial tumour recurrence. For patients without specific prognostic events, data were censored at the date of the last follow-up examination. Only patients with an OS and DFS of at least 3 months were included since early recurrence led to different therapeutic strategies in our cohort.

To identify predictive factors for OS and DFS, a Cox proportional hazards model was used. Data were presented as hazard ratios (HR) with 95% confidence intervals (95% CI). This was followed by the Cox multivariate model which included variables with *p* < 0.1 on univariate analysis for a further multivariate analysis. Due to incomplete documentation, statistical calculations for CEA values were only done with univariate analysis. A *p* value of < 0.05 was considered statistically significant. Kaplan–Meier curves were calculated using GraphPad Prism (Version 7, GraphPad Software, Inc., San Diego, USA), whereas univariate and multivariate analysis were performed using SPSS Statistics (Version 25, IBM, Armonk, NY, USA).

### Ethical approval

To assess the raw data from the Department of Surgery at the University Hospital of Wuerzburg approval from the ethics committee of the University of Wuerzburg was granted (Reference: 34/16). Written informed consent for resection surgery was obtained from all patients.

## Results

### Patient characteristics

**Fig. 1:**
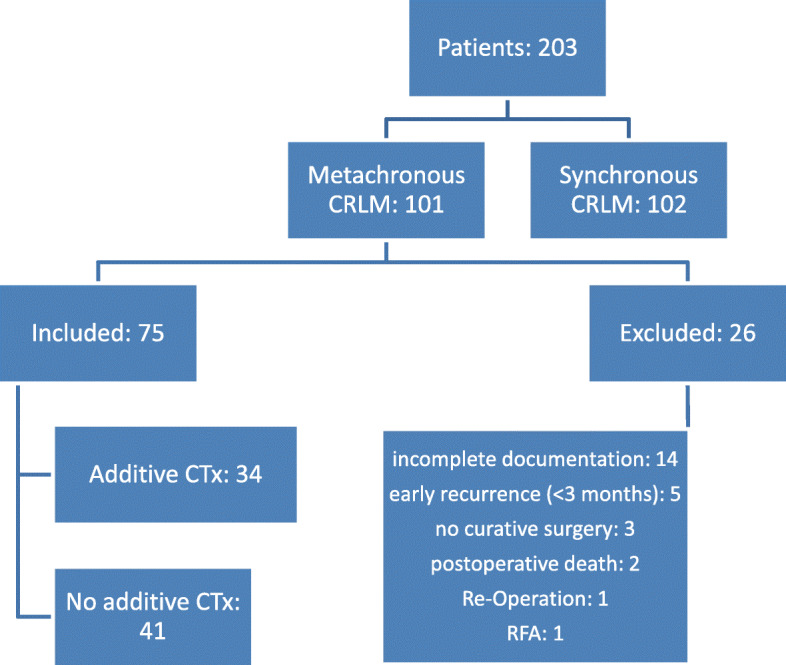
Study design

Seventy-five patients were finally included in this retrospective analysis including 27 female (36.0%) and 48 male (64.0%) patients (Fig. 1). As shown in Table 1, 44 patients (58.7%) were initially diagnosed with colon cancer as primary tumour and 31 patients with rectal cancer (41.3%). In the colon cancer group, 14 tumours (31.8%) were located in the right hemicolon and 30 tumours (68.2%) in the left hemicolon. The nodal status of the primary tumour was positive in 43 patients (54.1%). EM-CRLM were observed in 28 patients (36.5%) compared to 47 patients with LM-CRLM (63.5%). The median CEA value before hepatic resection was 22.97 ng/ml. Regarding the number of hepatic metastases, 70 patients (93.3%) had less than five lesions whereas four patients (5.3%) had five or more CRLM. In addition, the size of the greatest CRLM exceeded 5 cm in nine patients (12.0%). Twenty-seven patients subsequently underwent minor liver resection (36.0%) compared to 48 patients with major liver resection (64.0%). Patients with R1/R2 resection were excluded from the study. No patients of our cohort underwent local ablation therapy or staged hepatectomy. Neoadjuvant CTx was administered in 13 patients (18.9%) and 34 patients received additive CTx (45.3%).

**Table 1:** Patient demographics and clinical characteristics

	Total (***n*** = 75)		Resection only (***n*** = 41)		Resection plus additive CTx (***n*** = 34)		***p*** value
	n	%	n	%	n	%	
Age, years							
Median	65		65		65		ns
Range	35–85		42–84		35–85		
Sex							
Male	48	64.0	25	61.0	23	67.6	ns
Female	27	36.0	16	39.0	11	32.4	
Primary tumor							
Colon	44	58.7	23	56.1	21	61.8	ns
Right	14	31.8	4	17.4	10	47.6	
Left	30	68.2	19	82.6	11	52.4	
Rectum	31	41.3	18	43.9	13	38.2	
Primary nodal status							
N0	31	45.9	14	34.1	17	50.0	ns
N1/2	43	54.1	27	65.9	16	47.1	
Time between primary and hepatic resection							
< 12 months	28	36.5	14	34.1	14	39.4	ns
> 12 Months	47	63.5	27	65.9	20	60.6	
CEA before hepatic resection, ng/ml							
Median	22.97		22.86		23.12		ns
Range	1.1–150		1.6–150		1.1–137		
Number of CRLM							
< 5	70	93.3	38	92.7	32	88.2	ns
> 5	4	5.3	3	7.3	1	2.9	
Size of greatest CRLM							
< 5 cm	62	82.7	32	78.0	30	88.2	ns
> 5 cm	9	12.0	6	14.6	3	8.8	
Neoadjuvant CTx							
No	62	81.1	35	85.4	27	79.4	ns
Yes	13	18.9	6	14.6	7	20.6	
Surgical procedure							
Minor liver resection	27	36.0	16	39.0	11	32.4	ns
Major liver resection	48	64.0	25	61.0	23	67.6	

For survival analysis, we divided our cohort according to the administration of additive CTx following curative liver resection (Table 1). Thirty-four patients (45.3%) received postoperative chemotherapy whereas 41 patients (54.7%) underwent only resection. With regard to patient as well as tumour characteristics, we did not observe any significant differences between both groups.

### Evaluation of different variables on DFS and OS

Localization of the primary tumour, CEA serum levels and size of CRLM did not significantly affect DFS. In univariate analysis, a positive primary lymph node status (*p* = 0.034, HR 1.73, 95%-CI 1.04–2.88) as well as more than five CRLM (*p* = 0.005, HR 4.58, 95%-CI 1.58–13.26) were associated with a significantly decreased DFS. Concerning the time interval between curative treatment of the primary tumour and the occurrence of metachronous CRLM, there was a clear trend for an impaired DFS of patients diagnosed with EM-CRLM (DFS: 14 vs. 28 months; *p* = 0.11). In further multivariate analysis, primary lymph node status (*p* = 0.008, HR 2.01, 95%-CI 1.20–3.34) and the number of CRLM (*p* = 0.001, HR 6.49, 95%-CI 2.15–19.61) turned out to be independent prognostic factors for DFS (Table 2). No differences in terms of site of recurrence were seen between both groups.

**Table 2:** Univariate and multivariate analysis of DFS

	Number of patients	Median DFS, months	Univariate analysis			Multivariate analysis		
			***p*** value	HR	95% CI	***p*** value	HR	95% CI
Primary tumor								
Colon	44	36	0.14	1.53	0.87–2.68			
Rectum	31	18						
Primary nodal status								
N0	31	37	0.034	1.73	1.04–2.88	0.008	2.01	1.20–3.34
N1/2	43	14						
Time between primary and hepatic resection								
< 12 months	27	14	0.11	0.63	0.35–1.12			
> 12 months	48	28						
Number of CRLM								
< 5	70	28	0.005	4.58	1.58–13.26	0.001	6.49	2.15–19.61
> 5	4	7.5						
Size of greatest CRLM								
< 5 cm	62	20	0.27	1.62	0.68–3.82			
> 5 cm	9	29						
Neoadjuvant CTx								
No	62	20	0.62	0.83	0.39–1.76			
Yes	13	28						
Additive CTx								
No	41	18	0.29	0.74	0.42–1.29			
Yes	34	29						
CEA, ng/ml								
< 70	47	28	0.70	0.76	0.18–3.19			
> 70	5	19.5						

With regard to OS, localization and lymph nodes status of the primary tumour as well as size of CRLM did not significantly influence patient survival. Univariate analysis identified more than five CRLM (*p* = 0.012, HR 3.86, 95%-CI 1.35–11.05) and CEA serum levels higher than 70 ng/ml (*p* = 0.031, HR 4.24, 95%-CI 1.14–15.74) as risk factors for a significantly decreased OS. Furthermore, patients suffering from disease recurrence had a significantly worse prognostic outcome compared to patients without recurrence (*p* = 0.006, HR 2.65, 95% CI 1.32–5.36). In multivariate analysis, number of CRLM (*p* = 0.026, HR 3.46, 95%-CI 1.16–10.32), disease recurrence (*p* = 0.009, HR 2.70, 95% CI 1.29–5.65) and administration of additive chemotherapy (*p* = 0.016, HR 0.44, 95%-CI 0.23–0.86) were identified as independent prognostic factors (Table 3).

**Table 3:** Univariate and multivariate analysis of OS

	Number of patients	Median OS, months	Univariate analysis			Multivariate analysis		
			***p*** value	HR	95% CI	***p*** value	HR	95% CI
Primary tumor								
Colon	44	62	0.58	1.18	0.65–2.18			
Rectum	31	53						
Primary nodal status								
N0	31	65	0.16	1.50	0.86–2.64			
N1/2	43	51						
Time between primary and hepatic resection								
< 12 months	27	57	0.18	0.65	0.34–1.23			
> 12 months	48	75						
Number of CRLM								
< 5	70	62	0.012	3.86	1.35–11.05	0.026	3.46	1.16–10.32
> 5	4	19						
Size of greatest CRLM								
< 5 cm	62	62	0.21	1.83	0.71–4.71			
> 5 cm	9	27						
Neoadjuvant CTx								
No	62	62	0.30	0.63	0.26–1.51			
Yes	13	68						
Additive CTx								
No	41	57	0.032	0.50	0.26–0.94	0.016	0.44	0.23–0.86
Yes	34	62						
CEA, ng/ml								
< 70	47	65	0.031	4.24	1.14–15.74			
> 70	5	22						
Disease recurrence								
Yes	48	51	0.006	2.65	1.32–5.36	0.009	2.70	1.29–5.65
No	27	110						

### Impact of additive chemotherapy on DFS and OS

CTx regimens were fluorouracil-based in all patients. Of 34 patients receiving additive chemotherapy, 18 patients were treated with FOLFOX/FOLFIRI, 10 patients with Capecitabine and 6 patients with CAPOX. No therapies were terminated due to side effects based on chemotherapy.

**Fig. 2:**
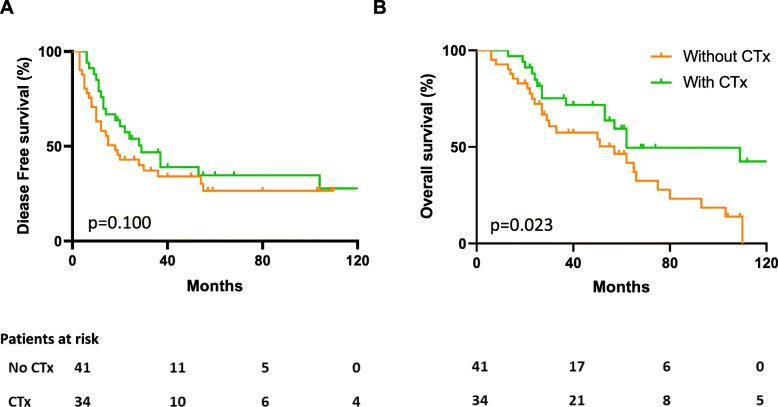
Impact of additive chemotherapy on disease-free and overall survival. DFS (**a**) and OS (**b**) analyzed by Kaplan-Meier with (green) and without (orange) additive CTx. Additive CTx resulted in a significantly prolonged OS (*p* = 0.023) in patients with metachronous CRLM

Administration of additive chemotherapy resulted in a prolonged median DFS (29 vs 18 months; *p* = 0.10) (Fig. 2a). In terms of OS, patients receiving additive CTx showed a significantly prolonged OS (62 vs 57 months, *p* = 0.023) (Fig. 2b). Additive chemotherapy significantly improved 10-year survival rates (42% vs 0%, *p* = 0.023), but not 5-year survival (58% vs 42%, *p* = 0.24).

## Discussion

Despite an increasing incidence of CRLM and a five-year postoperative survival of up to 70% with additive CTx in certain studies [25, 27], the value of perioperative CTx after curative resection of metachronous CRLM remains controversial [23, 26, 28, 29]. Therefore, we investigated the impact of applying additive CTx on the long-term outcome of patients with metachronous CRLM. In our patient cohort, the administration of additive CTx following R0 liver resection resulted in a significantly prolonged OS compared to patients without chemotherapy (*p* = 0.032). Furthermore, additive CTx was identified as independent risk factor for OS in multivariate analysis (*p* = 0.016, HR 0.44, 95% CI 0.23–0.86). Importantly, striking and statistically significant differences in OS were detected at later stages of follow-up with 10-year survival rates of 42% in the chemotherapy group compared to no long-term survivors in the resection-only group (*p* = 0.023).

Surgical resection as one part of a multimodal therapeutic concept remains the only curative treatment option for patients with CRLM. Treatment guidelines are well established for patients with synchronous CRLM [17]. However, consensus is lacking regarding perioperative treatment options for patients with metachronous CRLM. A beneficial role of perioperative CTx has been extensively discussed in the past and its significance has been differently weighted among medical societies. On the one hand, current guidelines of the National Comprehensive Cancer Networks (NCCN) in the United States clearly recommend additive CTx with FOLFOX or CAPOX for all patients with metachronous CRLM who had not received Oxaliplatin-based CTx preoperatively. Only if patients had obtained Oxaliplatin-based CTx earlier, postoperative observation is preferred [15, 16]. In contrast, European guidelines of the European Society for Medical Oncology (ESMO) recommend additive CTx only in case of unfavourable prognostic factors such as rapid tumour progression (i.e. short interval between resection of the primary tumour and the occurrence of metachronous CRLM), more than five liver metastasis or concomitant extrahepatic disease. If so, perioperative CTx is recommended with FOLFOX or CAPOX [17]. This therapeutic regimen has been accepted by several oncological societies in Asia [30]. However, current German guidelines do not recommend additive CTx [26].

Studies suggest that synchronous CRLM have a less favourable tumour biology and a worse prognostic outcome in comparison to metachronous CRLM [24, 25, 31]. Scientific evidence regarding the optimal postoperative treatment strategy in case of metachronous CRLM is lacking since most studies have focused on CRLM in general, thereby mixing patient cohorts suffering from synchronous or metachronous CRLM. A recent meta-analysis of three randomized trials detected a statistically significant prolonged DFS for patients with CRLM who had received additive CTx in comparison to patients without CTx. However, with regard to patients’ OS a calculation of the survival benefit after additive CTx missed the level of statistical significance closely [20, 29]. Other retrospective studies identified a survival advantage of additive CTx for certain patient subgroups. While Adam et al. demonstrated a significantly increased DFS and OS for patients with larger CRLM (> 5 cm) [32], another study showed a prognostic benefit of additional postoperative chemotherapy for patients with more than ten CRLM [22]. Nevertheless, both studies did not differentiate outcome data between patients with synchronous or metachronous CRLM. In a different approach, a recent Japanese and French multicentre study compared perioperative (neoadjuvant plus adjuvant) versus additive CTx with FOLFOX in the setting of CRLM including synchronous and metachronous metastases. Since they did not find a benefit for additional neoadjuvant CTx regarding DFS and OS, the authors recommend additive CTx only for patients with easily resectable CRLM [21]. In addition, a recent retrospective study by Nishioka et al. analysed the prognostic advantage of additive CTx on synchronous and metachronous CRLM. While no advantage was seen for late-metachronous CRLM (> 12 months), a significant prolonged OS and DFS for patients with synchronous and early-metachronous CRLM (< 12 months) was identified [25]. Thus, both studies support the approach of upfront surgery followed by additive CTx.

In this context, our results clearly promote the administration of additive CTx for patients after R0 resections of metachronous CRLM as OS was significantly increased in our patient cohort. This also applied for patients receiving neoadjuvant CTx prior to liver resection. Additive chemotherapy did not only prolong 5-year survival, but most impressively, it significantly improved 10-year survival rates (42% versus 0%). A systematic review about risk factors that influence ten-year survival after liver resection for colorectal liver metastases calculated a 10-year survival rate of 12–28% [33]. We might have detected better survival rates in our cohort due to a more stringent definition of patients as both, metachronous CRLM and R0-resections, are well-known factors for improved survival rates. Moreover, most of our patients were treated with more effective chemotherapeutic agents than 5-FU only as administered in older series [33]. Most importantly, our data emphasize the need for longer follow-up intervals. As most studies report actual 5-year survival statistics as evidence of an adequate oncologic outcome, we could show that at least in this selected patient cohort, a striking and statistically significant survival advantage becomes evident only after 5 years of follow up.

This study has several limitations including its retrospective character and the single-centre design. In addition, the variability of chemotherapeutic regimens narrows a statement about a favourable protocol. However, the long observation period with 10-year follow-up data is a major advantage for robust survival analysis.

To our knowledge, this is the first study focusing exclusively on patients with metachronous CRLM and a follow-up period of at least ten years. Metachronous CRLM most likely arise from persisting and circulating tumour cells after resection of the primary colorectal carcinoma. A prolonged OS following the administration of additive chemotherapy in our patient cohort might be a consequence of the eradication/containment of remnant malignant cells. While surgery only offers control of local disease, systemic therapy can devitalize disseminated malignant cells. As result, hepatic or extrahepatic disease recurrence might occur at later stages or not at all after additive CTx with subsequently prolonged OS as seen in our cohort. Furthermore, one major drawback of former studies in interpreting survival data has been the fact that patients with synchronous and metachronous CRLM were usually combined despite the less favourable tumour biology of synchronous CRLM. Thus, final conclusions about the role of additive CTx in patients with metachronous CRLM were limited [23, 26]. Therefore, our study sheds new light on a survival advantage of postoperative chemotherapy on patients with metachronous CRLM.

## Conclusions

In conclusion, the administration of additive CTx in patients following curative resection of metachronous CRLM might have great potential on the postoperative OS of patients. Subsequently, larger patient cohorts as well as multi-centre studies together with prospective randomized trials are necessary to confirm our results and to evaluate different chemotherapeutic regimens since there is currently no definite recommendation for this particular cohort. Close cooperation between oncologists, gastroenterologists and surgeons is necessary to provide optimal care for individual patients by developing sufficient additive treatment strategies following surgical resection.

## Data Availability

The datasets generated and/or analyzed during the current study are not publicly available due data safety protection but are available from the corresponding author on reasonable request.

## Abbreviations

CEA: Carcinoembryonic antigen
CRC: Colorectal cancer
CRLM: Colorectal liver metastases
CTx: Chemotherapy
DFS: Disease Free Survival
EM-CRLM: Early metachronous CRLM
FU: Fluorouracil
HR: Hazard ratio
LM-CRLM: Late metachronous CRLM
OS: Overall survival
